# (*E*)-2-(3,4-Dimeth­oxy­benzyl­idene)-5,6-dimeth­oxy-2,3-dihydro-1*H*-inden-1-one

**DOI:** 10.1107/S1600536810041619

**Published:** 2010-10-20

**Authors:** Mohamed Ashraf Ali, Rusli Ismail, Soo Choon Tan, Ching Kheng Quah, Hoong-Kun Fun

**Affiliations:** a Institute for Research in Molecular Medicine, Universiti Sains Malaysia, 11800 USM, Penang, Malaysia; b X-ray Crystallography Unit, School of Physics, Universiti Sains Malaysia, 11800 USM, Penang, Malaysia

## Abstract

In the title compound, C_20_H_20_O_5_, the 2,3-dihydro-1*H*-indene ring system is essentially planar [maximum deviation = 0.010 (1) Å] and is inclined at an angle of 4.09 (4)° with respect to the phenyl ring. The C=C bond has an *E* configuration. In the crystal, the mol­ecules are linked into chains propagating in [102] *via* inter­molecular C—H⋯O hydrogen bonds. The crystal structure is further consolidated by C—H⋯π inter­actions.

## Related literature

For general background to and the biological activity of chalcones, see: Nielsen *et al.* (1998 ▶); Go *et al.* (2005 ▶); Nowakowska (2007 ▶); Furusawa *et al.* (2005 ▶). For the stability of the temperature controller used in the data collection, see: Cosier & Glazer (1986 ▶). For bond-length data, see: Allen *et al.* (1987 ▶).
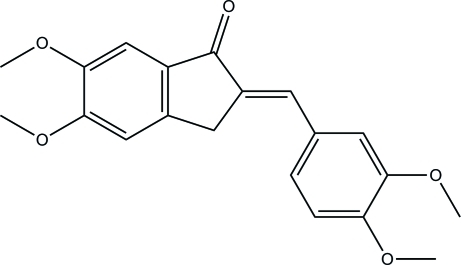


## Experimental

### 

#### Crystal data

C_20_H_20_O_5_
*M* *_r_* = 340.36
Monoclinic, 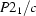

*a* = 7.7991 (7) Å
*b* = 7.2595 (6) Å
*c* = 29.589 (2) Å
β = 101.977 (3)°
*V* = 1638.8 (2) Å^3^
*Z* = 4
Mo *K*α radiation
μ = 0.10 mm^−1^
*T* = 100 K
0.53 × 0.45 × 0.09 mm

#### Data collection

Bruker APEXII DUO CCD diffractometer
Absorption correction: multi-scan (*SADABS*; Bruker, 2009 ▶) *T*_min_ = 0.950, *T*_max_ = 0.992
19303 measured reflections
5535 independent reflections
4471 reflections with *I* > 2σ(*I*)
*R*_int_ = 0.027

#### Refinement

*R*[*F* ^2^ > 2σ(*F* ^2^)] = 0.043
*wR*(*F* ^2^) = 0.124
*S* = 1.04
5535 reflections
230 parameters
H-atom parameters constrained
Δρ_max_ = 0.48 e Å^−3^
Δρ_min_ = −0.21 e Å^−3^

### 

Data collection: *APEX2* (Bruker, 2009 ▶); cell refinement: *SAINT* (Bruker, 2009 ▶); data reduction: *SAINT*; program(s) used to solve structure: *SHELXTL* (Sheldrick, 2008 ▶); program(s) used to refine structure: *SHELXTL*; molecular graphics: *SHELXTL*; software used to prepare material for publication: *SHELXTL* and *PLATON* (Spek, 2009 ▶).

## Supplementary Material

Crystal structure: contains datablocks global, I. DOI: 10.1107/S1600536810041619/hb5680sup1.cif

Structure factors: contains datablocks I. DOI: 10.1107/S1600536810041619/hb5680Isup2.hkl

Additional supplementary materials:  crystallographic information; 3D view; checkCIF report

## Figures and Tables

**Table 1:** Hydrogen-bond geometry (Å, °) *Cg*1 is the cetroid of C2–C7 benzene ring.

*D*—H⋯*A*	*D*—H	H⋯*A*	*D*⋯*A*	*D*—H⋯*A*
C18—H18*A*⋯O4^i^	0.96	2.34	3.0939 (13)	135
C1—H1*A*⋯*Cg*1^ii^	0.97	2.64	3.4804 (11)	146

Symmetry codes: (i) 
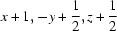
; (ii) 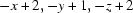.

**Fig. 1.**
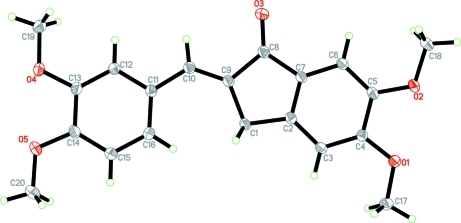
The molecular structure of the title compound showing 50% probability displacement ellipsoids for non-H atoms.

**Fig. 2.**
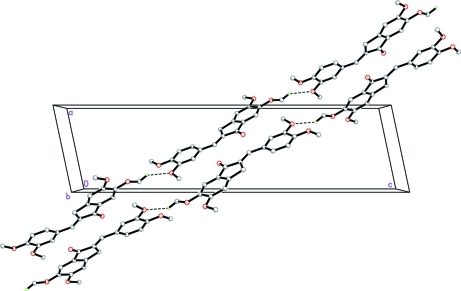
The crystal structure of the title compound, viewed along the b axis. H atoms not involved in hydrogen bonds (dashed lines) have been omitted for clarity.

## supplementary crystallographic information

### Comment

Chalcones are a chemical class that has shown promising therapeutic efficacy for the management of several diseases. Many papers have been presented in the literature with references to structural modifications of the chalcone template (Nielsen *et al.*, 1998). In fact, not many other structural templates can claim association with such a diverse range of pharmacological activities, among which cytotoxicity, antitumour, anti-inflammatory, antiplasmodial, immunosuppression and antioxidant, are widely cited (Go *et al.*, 2005). They considered as the precursor of flavonoids and isoflavonoids. Chemically they consisted of open chain flavonoid by a three carbon α, β-unsaturated carbonyl system (Nowakowska, 2007). In fact, the pharmacological properties of chalcones are due to the presence of both α, β-unsaturation (Furusawa *et al.*, 2005) and an aromatic ring.

In the title molecule (Fig. 1), the 2,3-dihydro-1*H*-indene (C1-C9) ring system is essentially planar (maximum deviation = 0.010 (1) Å for atom C7) and is inclined at an angle of 4.09 (4) ° with the phenyl ring (C11-C16), which indicates they are almost parallel to each other. The crystal structure determination shows the *E* configuration of the C9═C10 and confirms that the molecule adopts an overall planar conformation, with the exception of the methyl moieties. Bond lengths (Allen *et al.*, 1987) and angles are within normal ranges.

In the solid state (Fig. 2), the molecules are linked into one-dimensional chains along [102] *via* intermolecular C18–H18A···O4 hydrogen bonds (Table 1). The crystal structure are further consolidated by C–H···π (Table 1) interactions.

### Experimental

A mixture of 5,6-dimethoxy-2,3-dihydro-1*H*-indene-1-one (0.001 mmol) and 3,4-dimethoxy benzaldehyde (0.001 mmol) were dissolved in methanol (10 mL) and 30% sodium hydroxide solution (5 mL) was added and stirred for 5 h. After completion of the reaction as evident from TLC (thin layer chromatography), the mixture was poured into crushed ice then neutralized with concentrated HCl. The precipitated solid was filtered, washed with water and recrystallised from ethanol to reveal yellow plates of (I).

### Refinement

All H atoms were positioned geometrically and refined using a riding model with C–H = 0.93-0.97 Å and *U*_iso_(H) = 1.2 or 1.5 *U*_eq_(C). A rotating-group model was applied for the methyl groups. The highest residual electron density peak is located at 0.69 Å from C6 and the deepest hole is located at 1.13 Å from C7.

### Crystal data

**Table:**

C_20_H_20_O_5_	*F*(000) = 720
*M**_r_* = 340.36	*D*_x_ = 1.380 Mg m^−^^3^
Monoclinic, *P*2_1_/*c*	Mo *K*α radiation, λ = 0.71073 Å
Hall symbol: -P 2ybc	Cell parameters from 6986 reflections
*a* = 7.7991 (7) Å	θ = 2.7–31.7°
*b* = 7.2595 (6) Å	µ = 0.10 mm^−^^1^
*c* = 29.589 (2) Å	*T* = 100 K
β = 101.977 (3)°	Plate, yellow
*V* = 1638.8 (2) Å^3^	0.53 × 0.45 × 0.09 mm
*Z* = 4	

### Data collection

**Table:**

Bruker APEXII DUO CCD diffractometer	5535 independent reflections
Radiation source: fine-focus sealed tube	4471 reflections with *I* > 2σ(*I*)
graphite	*R*_int_ = 0.027
φ and ω scans	θ_max_ = 31.8°, θ_min_ = 2.7°
Absorption correction: multi-scan (*SADABS*; Bruker, 2009)	*h* = −11→11
*T*_min_ = 0.950, *T*_max_ = 0.992	*k* = −10→10
19303 measured reflections	*l* = −41→43

### Refinement

**Table:**

Refinement on *F*^2^	Primary atom site location: structure-invariant direct methods
Least-squares matrix: full	Secondary atom site location: difference Fourier map
*R*[*F*^2^ > 2σ(*F*^2^)] = 0.043	Hydrogen site location: inferred from neighbouring sites
*wR*(*F*^2^) = 0.124	H-atom parameters constrained
*S* = 1.04	*w* = 1/[σ^2^(*F*_o_^2^) + (0.0668*P*)^2^ + 0.3686*P*] where *P* = (*F*_o_^2^ + 2*F*_c_^2^)/3
5535 reflections	(Δ/σ)_max_ = 0.002
230 parameters	Δρ_max_ = 0.48 e Å^−^^3^
0 restraints	Δρ_min_ = −0.21 e Å^−^^3^

### Special details

**Table:**

Experimental. The crystal was placed in the cold stream of an Oxford Cyrosystems Cobra open-flow nitrogen cryostat (Cosier & Glazer, 1986) operating at 100.0 (1) K.
Geometry. All esds (except the esd in the dihedral angle between two l.s. planes) are estimated using the full covariance matrix. The cell esds are taken into account individually in the estimation of esds in distances, angles and torsion angles; correlations between esds in cell parameters are only used when they are defined by crystal symmetry. An approximate (isotropic) treatment of cell esds is used for estimating esds involving l.s. planes.
Refinement. Refinement of F^2^ against ALL reflections. The weighted R-factor wR and goodness of fit S are based on F^2^, conventional R-factors R are based on F, with F set to zero for negative F^2^. The threshold expression of F^2^ > 2sigma(F^2^) is used only for calculating R-factors(gt) etc. and is not relevant to the choice of reflections for refinement. R-factors based on F^2^ are statistically about twice as large as those based on F, and R- factors based on ALL data will be even larger.

### Fractional atomic coordinates and isotropic or equivalent isotropic displacement parameters (Å^2^)

**Table:**

	*x*	*y*	*z*	*U*_iso_*/*U*_eq_	
O1	1.13991 (11)	0.89751 (10)	1.10930 (2)	0.02109 (16)	
O2	1.10936 (10)	0.63257 (10)	1.16292 (2)	0.02089 (16)	
O3	0.67192 (11)	0.17121 (11)	1.04585 (3)	0.02565 (18)	
O4	0.20749 (12)	0.09725 (11)	0.79852 (3)	0.02789 (19)	
O5	0.29534 (11)	0.38568 (11)	0.75804 (2)	0.02474 (18)	
C1	0.73939 (12)	0.57231 (13)	0.97826 (3)	0.01539 (17)	
H1A	0.8144	0.5695	0.9559	0.018*	
H1B	0.6512	0.6672	0.9695	0.018*	
C2	0.84490 (12)	0.60464 (13)	1.02641 (3)	0.01469 (17)	
C3	0.94756 (13)	0.75715 (13)	1.04280 (3)	0.01598 (18)	
H3A	0.9571	0.8557	1.0234	0.019*	
C4	1.03547 (13)	0.75888 (13)	1.08879 (3)	0.01591 (18)	
C5	1.01914 (13)	0.60926 (13)	1.11875 (3)	0.01583 (18)	
C6	0.91645 (13)	0.45957 (13)	1.10221 (3)	0.01591 (17)	
H6A	0.9045	0.3613	1.1215	0.019*	
C7	0.83024 (12)	0.45901 (13)	1.05551 (3)	0.01495 (17)	
C8	0.71294 (12)	0.31778 (13)	1.03029 (3)	0.01670 (18)	
C9	0.65574 (12)	0.38678 (13)	0.98188 (3)	0.01564 (18)	
C10	0.54838 (12)	0.28635 (13)	0.94942 (3)	0.01632 (18)	
H10A	0.5090	0.1764	0.9598	0.020*	
C11	0.48499 (12)	0.32517 (13)	0.90031 (3)	0.01607 (18)	
C12	0.37662 (13)	0.19248 (13)	0.87386 (3)	0.01689 (18)	
H12A	0.3464	0.0866	0.8881	0.020*	
C13	0.31431 (14)	0.21748 (14)	0.82688 (3)	0.01865 (19)	
C14	0.36029 (13)	0.37598 (14)	0.80457 (3)	0.01844 (19)	
C15	0.46461 (14)	0.50818 (14)	0.83057 (3)	0.0205 (2)	
H15A	0.4942	0.6143	0.8163	0.025*	
C16	0.52578 (14)	0.48376 (14)	0.87797 (3)	0.0202 (2)	
H16A	0.5948	0.5746	0.8950	0.024*	
C17	1.16649 (16)	1.04933 (14)	1.08102 (4)	0.0240 (2)	
H17A	1.2447	1.1362	1.0991	0.036*	
H17B	1.0562	1.1080	1.0689	0.036*	
H17C	1.2165	1.0061	1.0559	0.036*	
C18	1.10498 (16)	0.48297 (16)	1.19368 (3)	0.0249 (2)	
H18A	1.1819	0.5085	1.2228	0.037*	
H18B	1.1425	0.3725	1.1808	0.037*	
H18C	0.9876	0.4669	1.1983	0.037*	
C19	0.14395 (17)	−0.05676 (16)	0.81966 (4)	0.0273 (2)	
H19A	0.0667	−0.1276	0.7966	0.041*	
H19B	0.0813	−0.0147	0.8424	0.041*	
H19C	0.2408	−0.1322	0.8343	0.041*	
C20	0.34068 (19)	0.54410 (17)	0.73445 (4)	0.0317 (3)	
H20A	0.2863	0.5362	0.7023	0.048*	
H20B	0.4656	0.5496	0.7377	0.048*	
H20C	0.3006	0.6530	0.7475	0.048*	

### Atomic displacement parameters (Å^2^)

**Table:**

	*U*^11^	*U*^22^	*U*^33^	*U*^12^	*U*^13^	*U*^23^
O1	0.0295 (4)	0.0158 (3)	0.0161 (3)	−0.0051 (3)	0.0005 (3)	0.0003 (2)
O2	0.0289 (4)	0.0214 (3)	0.0106 (3)	−0.0031 (3)	−0.0002 (3)	0.0017 (2)
O3	0.0300 (4)	0.0223 (4)	0.0217 (3)	−0.0077 (3)	−0.0016 (3)	0.0067 (3)
O4	0.0412 (5)	0.0264 (4)	0.0150 (3)	−0.0166 (3)	0.0035 (3)	−0.0035 (3)
O5	0.0322 (4)	0.0277 (4)	0.0128 (3)	−0.0068 (3)	0.0013 (3)	0.0024 (3)
C1	0.0150 (4)	0.0183 (4)	0.0122 (3)	−0.0007 (3)	0.0014 (3)	0.0022 (3)
C2	0.0135 (4)	0.0174 (4)	0.0133 (4)	0.0013 (3)	0.0030 (3)	0.0009 (3)
C3	0.0189 (4)	0.0156 (4)	0.0132 (4)	0.0000 (3)	0.0029 (3)	0.0019 (3)
C4	0.0183 (4)	0.0147 (4)	0.0144 (4)	−0.0004 (3)	0.0026 (3)	−0.0006 (3)
C5	0.0181 (4)	0.0177 (4)	0.0114 (3)	0.0018 (3)	0.0022 (3)	0.0005 (3)
C6	0.0172 (4)	0.0168 (4)	0.0136 (4)	0.0004 (3)	0.0029 (3)	0.0022 (3)
C7	0.0143 (4)	0.0166 (4)	0.0136 (4)	0.0002 (3)	0.0021 (3)	0.0016 (3)
C8	0.0147 (4)	0.0189 (4)	0.0158 (4)	−0.0005 (3)	0.0017 (3)	0.0018 (3)
C9	0.0146 (4)	0.0179 (4)	0.0139 (4)	−0.0001 (3)	0.0018 (3)	0.0016 (3)
C10	0.0152 (4)	0.0181 (4)	0.0153 (4)	−0.0006 (3)	0.0025 (3)	0.0009 (3)
C11	0.0149 (4)	0.0183 (4)	0.0149 (4)	−0.0005 (3)	0.0027 (3)	−0.0006 (3)
C12	0.0196 (4)	0.0171 (4)	0.0146 (4)	−0.0019 (3)	0.0049 (3)	−0.0007 (3)
C13	0.0219 (5)	0.0188 (4)	0.0155 (4)	−0.0041 (4)	0.0045 (3)	−0.0035 (3)
C14	0.0198 (4)	0.0216 (4)	0.0135 (4)	−0.0005 (4)	0.0026 (3)	0.0002 (3)
C15	0.0224 (5)	0.0203 (4)	0.0176 (4)	−0.0035 (4)	0.0016 (4)	0.0031 (3)
C16	0.0211 (4)	0.0205 (4)	0.0174 (4)	−0.0057 (4)	0.0001 (3)	0.0005 (3)
C17	0.0334 (6)	0.0151 (4)	0.0219 (4)	−0.0044 (4)	0.0020 (4)	0.0017 (3)
C18	0.0312 (5)	0.0270 (5)	0.0147 (4)	−0.0029 (4)	0.0009 (4)	0.0066 (4)
C19	0.0366 (6)	0.0237 (5)	0.0228 (5)	−0.0125 (5)	0.0091 (4)	−0.0038 (4)
C20	0.0415 (7)	0.0325 (6)	0.0191 (5)	−0.0071 (5)	0.0013 (5)	0.0088 (4)

### Geometric parameters (Å, °)

**Table:**

O1—C4	1.3562 (11)	C10—C11	1.4618 (12)
O1—C17	1.4247 (12)	C10—H10A	0.9300
O2—C5	1.3601 (11)	C11—C16	1.3968 (14)
O2—C18	1.4220 (12)	C11—C12	1.4072 (13)
O3—C8	1.2279 (12)	C12—C13	1.3862 (13)
O4—C13	1.3674 (12)	C12—H12A	0.9300
O4—C19	1.4197 (13)	C13—C14	1.4094 (14)
O5—C14	1.3667 (11)	C14—C15	1.3839 (14)
O5—C20	1.4273 (14)	C15—C16	1.3958 (13)
C1—C2	1.5084 (12)	C15—H15A	0.9300
C1—C9	1.5100 (14)	C16—H16A	0.9300
C1—H1A	0.9700	C17—H17A	0.9600
C1—H1B	0.9700	C17—H17B	0.9600
C2—C7	1.3832 (13)	C17—H17C	0.9600
C2—C3	1.3932 (13)	C18—H18A	0.9600
C3—C4	1.3909 (12)	C18—H18B	0.9600
C3—H3A	0.9300	C18—H18C	0.9600
C4—C5	1.4244 (13)	C19—H19A	0.9600
C5—C6	1.3781 (13)	C19—H19B	0.9600
C6—C7	1.4057 (12)	C19—H19C	0.9600
C6—H6A	0.9300	C20—H20A	0.9600
C7—C8	1.4703 (13)	C20—H20B	0.9600
C8—C9	1.4955 (12)	C20—H20C	0.9600
C9—C10	1.3497 (13)		
			
C4—O1—C17	117.28 (7)	C13—C12—C11	120.88 (9)
C5—O2—C18	116.34 (8)	C13—C12—H12A	119.6
C13—O4—C19	117.15 (8)	C11—C12—H12A	119.6
C14—O5—C20	117.16 (8)	O4—C13—C12	125.03 (9)
C2—C1—C9	103.34 (7)	O4—C13—C14	114.56 (8)
C2—C1—H1A	111.1	C12—C13—C14	120.41 (9)
C9—C1—H1A	111.1	O5—C14—C15	125.21 (9)
C2—C1—H1B	111.1	O5—C14—C13	115.89 (9)
C9—C1—H1B	111.1	C15—C14—C13	118.89 (9)
H1A—C1—H1B	109.1	C14—C15—C16	120.63 (9)
C7—C2—C3	120.42 (8)	C14—C15—H15A	119.7
C7—C2—C1	111.71 (8)	C16—C15—H15A	119.7
C3—C2—C1	127.88 (8)	C15—C16—C11	121.08 (9)
C4—C3—C2	118.61 (8)	C15—C16—H16A	119.5
C4—C3—H3A	120.7	C11—C16—H16A	119.5
C2—C3—H3A	120.7	O1—C17—H17A	109.5
O1—C4—C3	124.94 (8)	O1—C17—H17B	109.5
O1—C4—C5	114.23 (8)	H17A—C17—H17B	109.5
C3—C4—C5	120.82 (8)	O1—C17—H17C	109.5
O2—C5—C6	125.87 (9)	H17A—C17—H17C	109.5
O2—C5—C4	114.07 (8)	H17B—C17—H17C	109.5
C6—C5—C4	120.05 (8)	O2—C18—H18A	109.5
C5—C6—C7	118.39 (9)	O2—C18—H18B	109.5
C5—C6—H6A	120.8	H18A—C18—H18B	109.5
C7—C6—H6A	120.8	O2—C18—H18C	109.5
C2—C7—C6	121.70 (9)	H18A—C18—H18C	109.5
C2—C7—C8	109.88 (8)	H18B—C18—H18C	109.5
C6—C7—C8	128.41 (9)	O4—C19—H19A	109.5
O3—C8—C7	126.65 (8)	O4—C19—H19B	109.5
O3—C8—C9	126.89 (9)	H19A—C19—H19B	109.5
C7—C8—C9	106.45 (8)	O4—C19—H19C	109.5
C10—C9—C8	121.23 (9)	H19A—C19—H19C	109.5
C10—C9—C1	130.15 (8)	H19B—C19—H19C	109.5
C8—C9—C1	108.62 (7)	O5—C20—H20A	109.5
C9—C10—C11	129.43 (9)	O5—C20—H20B	109.5
C9—C10—H10A	115.3	H20A—C20—H20B	109.5
C11—C10—H10A	115.3	O5—C20—H20C	109.5
C16—C11—C12	118.07 (8)	H20A—C20—H20C	109.5
C16—C11—C10	124.47 (8)	H20B—C20—H20C	109.5
C12—C11—C10	117.45 (9)		
			
C9—C1—C2—C7	0.61 (11)	C7—C8—C9—C10	178.86 (9)
C9—C1—C2—C3	−179.23 (10)	O3—C8—C9—C1	−179.52 (10)
C7—C2—C3—C4	0.46 (15)	C7—C8—C9—C1	−0.30 (11)
C1—C2—C3—C4	−179.72 (9)	C2—C1—C9—C10	−179.22 (10)
C17—O1—C4—C3	−2.52 (15)	C2—C1—C9—C8	−0.16 (10)
C17—O1—C4—C5	178.15 (9)	C8—C9—C10—C11	−176.82 (10)
C2—C3—C4—O1	179.82 (9)	C1—C9—C10—C11	2.15 (18)
C2—C3—C4—C5	−0.89 (15)	C9—C10—C11—C16	−1.16 (17)
C18—O2—C5—C6	4.19 (15)	C9—C10—C11—C12	178.32 (10)
C18—O2—C5—C4	−176.80 (9)	C16—C11—C12—C13	0.89 (15)
O1—C4—C5—O2	0.83 (13)	C10—C11—C12—C13	−178.62 (9)
C3—C4—C5—O2	−178.53 (9)	C19—O4—C13—C12	5.66 (17)
O1—C4—C5—C6	179.91 (9)	C19—O4—C13—C14	−174.51 (10)
C3—C4—C5—C6	0.55 (15)	C11—C12—C13—O4	−179.43 (10)
O2—C5—C6—C7	179.19 (9)	C11—C12—C13—C14	0.74 (16)
C4—C5—C6—C7	0.23 (15)	C20—O5—C14—C15	0.73 (16)
C3—C2—C7—C6	0.33 (15)	C20—O5—C14—C13	−179.60 (10)
C1—C2—C7—C6	−179.52 (9)	O4—C13—C14—O5	−1.25 (14)
C3—C2—C7—C8	179.02 (9)	C12—C13—C14—O5	178.59 (10)
C1—C2—C7—C8	−0.83 (11)	O4—C13—C14—C15	178.44 (10)
C5—C6—C7—C2	−0.67 (15)	C12—C13—C14—C15	−1.72 (16)
C5—C6—C7—C8	−179.10 (10)	O5—C14—C15—C16	−179.29 (10)
C2—C7—C8—O3	179.92 (10)	C13—C14—C15—C16	1.05 (16)
C6—C7—C8—O3	−1.51 (18)	C14—C15—C16—C11	0.60 (17)
C2—C7—C8—C9	0.70 (11)	C12—C11—C16—C15	−1.57 (16)
C6—C7—C8—C9	179.27 (10)	C10—C11—C16—C15	177.91 (10)
O3—C8—C9—C10	−0.35 (17)		

### Hydrogen-bond geometry (Å, °)

**Table:**

Cg1 is the cetroid of C2–C7 benzene ring.

**Table:**

*D*—H···*A*	*D*—H	H···*A*	*D*···*A*	*D*—H···*A*
C18—H18A···O4^i^	0.96	2.34	3.0939 (13)	135
C1—H1A···Cg1^ii^	0.97	2.64	3.4804 (11)	146

Symmetry codes: (i) *x*+1, −*y*+1/2, *z*+1/2; (ii) −*x*+2, −*y*+1, −*z*+2.

## References

[bb1] Allen, F. H., Kennard, O., Watson, D. G., Brammer, L., Orpen, A. G. & Taylor, R. (1987). *J. Chem. Soc. Perkin Trans. 2*, pp. S1–S19.

[bb2] Bruker (2009). *APEX2*, *SAINT* and *SADABS* Bruker AXS Inc., Madison, Wisconsin, USA.

[bb3] Cosier, J. & Glazer, A. M. (1986). *J. Appl. Cryst.* **19**, 105–107.

[bb4] Furusawa, M., Tanaka, T., Ito, T., Nishikawa, A., Yamazaki, N., Nakaya, K., Matsuura, N., Tsuchiya, H., Nagayama, M. & Iinuma, M. (2005). *J. Health Sci.* **51**, 376–378.

[bb5] Go, M. L., Wu, X. & Liu, X. L. (2005). *Curr. Med. Chem.* **12**, 483–499.

[bb6] Nielsen, S. B., Christensen, S. F., Cruciani, G. & Kharazmi, A. (1998). *J. Med. Chem.* **41**, 4819–4832 10.1021/jm980410m 9822551

[bb7] Nowakowska, Z. (2007). *Eur. J. Med. Chem.* **42**, 125–137. 10.1016/j.ejmech.2006.09.019 17112640

[bb8] Sheldrick, G. M. (2008). *Acta Cryst.* A**64**, 112–122. 10.1107/S0108767307043930 18156677

[bb9] Spek, A. L. (2009). *Acta Cryst.* D**65**, 148–155. 10.1107/S090744490804362X PMC2631630 19171970

